# Comparison of Molecular Recognition of Trimethyllysine and Trimethylthialysine by Epigenetic Reader Proteins

**DOI:** 10.3390/molecules25081918

**Published:** 2020-04-21

**Authors:** Jordi C. J. Hintzen, Jordi Poater, Kiran Kumar, Abbas H. K. Al Temimi, Bas J. G. E. Pieters, Robert S. Paton, F. Matthias Bickelhaupt, Jasmin Mecinović

**Affiliations:** 1 Department of Physics, Chemistry and Pharmacy, University of Southern Denmark, Campusvej 55, 5230 Odense, Denmark; 2 ICREA and Departament de Química Inorgànica i Orgànica & IQTCUB, Universitat de Barcelona, Martí I Franquès 1–11, 08028 Barcelona, Spain; 3 Chemistry Research Laboratory, University of Oxford, 12 Mansfield Road, Oxford OX1 3TA, UK; 4 Institute for Molecules and Materials, Radboud University, Heyendaalseweg 135, 6522 AJ Nijmegen, The Netherlands; 5 Department of Theoretical Chemistry and Amsterdam Center for Multiscale Modeling, Vrije Universiteit Amsterdam, De Boelelaan 1083, 1081HV Amsterdam, The Netherlands

**Keywords:** epigenetics, histone, lysine methylation, molecular recognition, noncovalent interactions

## Abstract

Gaining a fundamental insight into the biomolecular recognition of posttranslationally modified histones by epigenetic reader proteins is of crucial importance to understanding the regulation of the activity of human genes. Here, we seek to establish whether trimethylthialysine, a simple trimethyllysine analogue generated through cysteine alkylation, is a good trimethyllysine mimic for studies on molecular recognition by reader proteins. Histone peptides bearing trimethylthialysine and trimethyllysine were examined for binding with five human reader proteins employing a combination of thermodynamic analyses, molecular dynamics simulations and quantum chemical analyses. Collectively, our experimental and computational findings reveal that trimethylthialysine and trimethyllysine exhibit very similar binding characteristics for the association with human reader proteins, thereby justifying the use of trimethylthialysine for studies aimed at dissecting the origin of biomolecular recognition in epigenetic processes that play important roles in human health and disease.

## 1. Introduction

**Figure 1:**
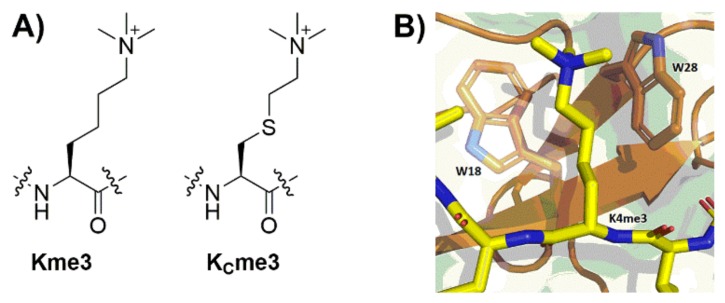
(**A**) Structures of of trimethyllysine (**Kme3**) and trimethylthialysine (**K_c_me3**); (**B**) view of the KDM5A_PHD3_ (orange) structure complexed with histone H3K4me3 (yellow) (PDB ID: 2KGI).

Biomolecular recognition of posttranslationally modified histone proteins is centrally important to regulation of the activity of human genes [1]. One of the most important and widespread histone modifications is lysine methylation, which is found on core histones and histone tails [2,3]. Histone lysine methyltransferases (KMTs) catalyze the transfer of methyl group from *S-*adenosylmethionine (SAM) to lysine ε-amino group, leading to three different methylation states (i.e., monomethyllysine Kme, dimethyllysine Kme2 and trimethyllysine Kme3, Figure 1A), which can be removed by histone lysine demethylases (KDMs) [4,5]. Methylated lysine residues play different roles in epigenetic processes, as these marks are specifically recognized by structurally diverse classes of epigenetic reader proteins. The highest mark, trimethyllysine, is recognized by the aromatic cage-containing readers, including tandem tudor domains (TTD), chromodomains (CD) and the plant homeodomain (PHD) zinc finger proteins (Figure 1B) [6]. To gain a better understanding of the exact role of lysine methylation in epigenetics, it is important to develop novel chemical tools for studying the molecular mechanisms that govern the molecular recognition of methylated lysines by reader proteins. An installation of chemically modified methylated lysine analogues into histone proteins [7] and histone peptides [8,9,10,11] has been a valuable method to study how changes in structure affect the association with reader proteins. In addition, a variety of methods have been developed to incorporate unnatural amino acids in both histone proteins and peptides, notably by auxotrophic expression systems [12] or employing the amber stop codon (TAG) [13]. Major drawbacks of both methods include severe limitations of amino acid variants that can be incorporated into histones. Furthermore, when using auxotrophic strains, the protein expression yield is decreased dramatically, and the process of developing an amber codon pair is time consuming and laborious [14,15].

Meanwhile, several synthetic and semi-synthetic methods have been developed to allow a site-specific incorporation of natural and unnatural amino acids in histones. Native chemical ligation can be used to develop fully synthetic proteins bearing the desired posttranslationally modified amino acids site-specifically [16]. This strategy has been shown to be viable in obtaining fully synthetic histone 3 [17]. Full protein synthesis can still be challenging and laborious, however, therefore, a desired alternative is found in synthetically simpler methods. The unique properties of the thiol group of cysteine have been used to this end. Cysteine can be selectively alkylated to obtain analogues that mimic naturally occurring amino acids. Among these are arginine [18], lysine [19,20] and different posttranslationally modified variants of lysine, including acetylated [21] and methylated analogues [22]. For the purpose of generating methylated lysine, simple bromides can be used for chemoselective reaction with the cysteine thiol to obtain intact histone proteins that possess simplest methylated lysine analogues (MLAs) [23].

Cysteine alkylation is an especially valuable method for examinations of histones, as only one or two native cysteine residues exist in four histone proteins; mutating C110 in H3 into an alanine (C110A) does not lead to any loss of function [22]. It has been shown that trimethylthialysine (K_C_me3, Figure 1A), the alkylated cysteine analogue of trimethyllysine, is well recognized by different epigenetic reader proteins [24], but as of yet, it is still up for debate whether the thioether bond created in the backbone of the trimethylthialysine is actually a good way to mimic the natural C–C bond in trimethyllysine, and somewhat conflicting reports in literature have surfaced [24,25]. When comparing the C–C bond to the C–S bond present in these analogues, it is evident that there are some differences in the bond angle (C–S–C is about 12 degrees smaller than C–C–C), while the C–S bond length is about 20% longer than the length of the C–C bond [25]. These two factors counteract each other to some extent, but still on average there is ~0.3 Å expansion of the distance between the geminal methylene units in the alkane and the thioether variant. Whether these subtle alterations contribute to differences in molecular recognition of methylated lysines by reader proteins seems to be heavily sequence and binder dependent, as there have been reports on practically no loss of binding affinity to 13-fold decreases [24,25]. The objective of this work is to systematically compare the origin of molecular recognition of trimethyllysine and trimethylthialysine-possessing histones by reader proteins, using a combination of thermodynamic analyses, molecular dynamics simulations and quantum chemical analyses.

## 2. Results

**Figure 2:**
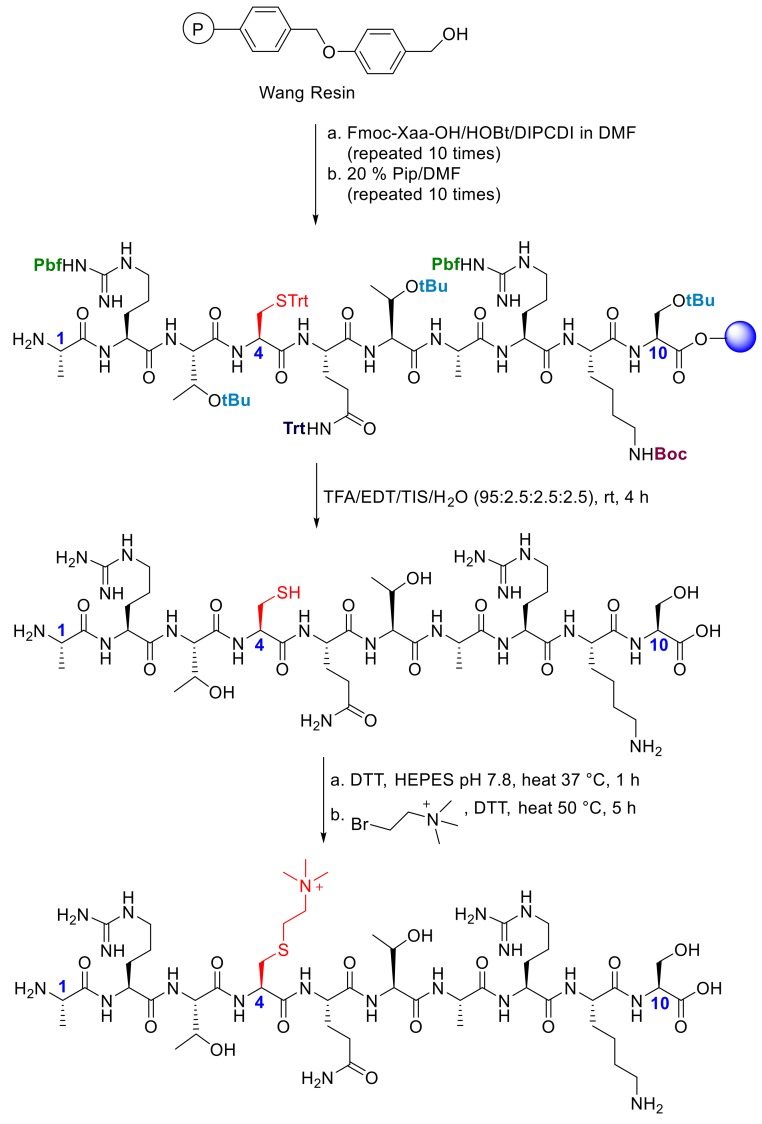
Solid-phase peptide synthesis of histone peptide H3K_C_4me3.

We synthesized 10-mer histone H3 peptides that possessed natural trimethyllysine (H3K4me3) and unnatural trimethylthialysine (H3K_C_4me3) by solid-phase peptide synthesis and purified them by preparative HPLC (Figure 2 and Scheme S1). A cysteine residue was introduced at position 4 of the histone peptide, which was site-specifically reacted with (2-bromoethyl)trimethylammonium bromide to form trimethylthialysine. Both synthetic histone peptides were purified by preparative HPLC.

We then examined both peptides for association with five human reader proteins (KDM5A_PHD3_, PDB: 2KGI; TAF3_PHD_, PDB: 2K17; BPTF_PHD_, PDB: 2F6J; SGF29_TTD_, PDB: 3ME9; KDM4A_TTD_, PDB: 2GFA) using isothermal titration calorimetry (ITC). This panel of epigenetic reader proteins has been characterized by means of structural determination and binding with H3K4me3, and provides a diverse composition and architecture of the aromatic cage [9]. ITC studies provided thermodynamic parameters (Gibbs free energy of binding Δ*G*°, enthalpy of binding Δ*H*°, entropy of binding Δ*S*°) for the association between five reader proteins and the two histone peptides (Table 1, Table S1 and Figure S1). We found that all examined reader proteins associate with the H3K_C_4me3 peptide with comparable dissociation constants to the natural H3K4me3 sequence, and that for both histone peptides the association with reader proteins is enthalpy-driven, a result that we attribute to the presence of several energetically favorable noncovalent interactions. In three cases, a slight decrease (~2-fold) in binding affinity was observed, whereas for TAF3_PHD_ and KDM4A_TTD_ a 2-fold increase in binding affinity was found. Differences in Δ*G*° were small, in the range of −0.5 to 0.5 kcal mol^−1^. In all cases, except for KDM4A_TTD_, it was observed that Δ*H*° was more unfavorable, while Δ*S*° is more favorable when going from H3K4me3 to H3K_C_4me3. With TAF3_PHD_, the gain in Δ*S*° is large enough to obtain an overall stronger binding affinity for H3K_C_4me3. These results are in agreement with earlier binding studies on different binding proteins for trimethyllysine and trimethylthialysine [24], and differ from recent findings showing that binding of BPTF_PHD_ leads to a much larger decrease in binding affinity (~13-fold) when comparing H3K4me3 and H3K_C_4me3 [25]. In support of the former observation, we found that even longer trimethylhomolysine is very well recognized by a panel of the same five reader proteins [10], indicating that the slightly longer C–S–C moiety (compared to C–C–C core) does not significantly alter the readout process, supporting the finding that BPTF_PHD_ indeed should recognize trimethylthialysine well.

**Figure 3:**
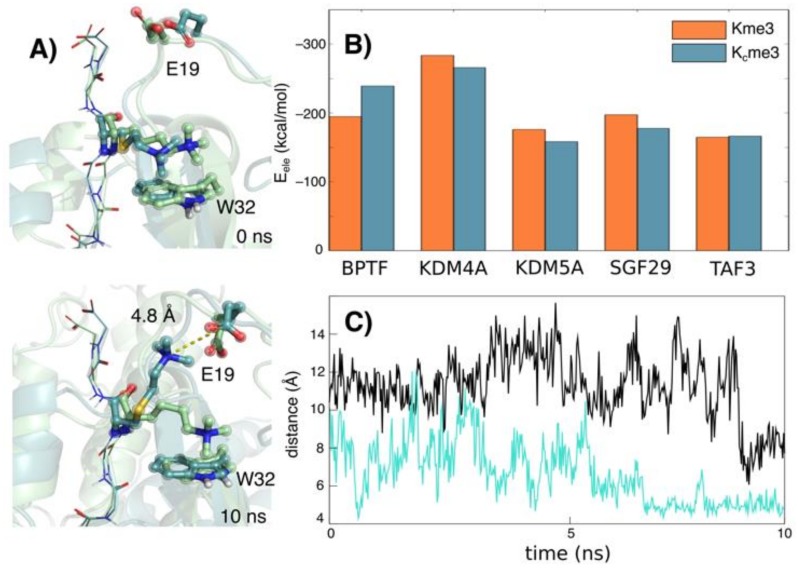
(**A**) Snapshots of BPTF_PHD_ complexed with H3 tail backbone (lines) containing K_C_me3 (cyan) and Kme3 (green) active sites at 0 and 10 ns; (**B**) ΔE_ele_ contributions of ligands Kme3 and K_C_me3 from MM-GBSA binding free energy calculations; (**C**) distance vs. time plot of side Nε^+^ atoms of Kme3 (black) and K_C_me3 (cyan) to carboxyl group of BPTF_PHD_ residue Glu19 (E19).

Following thermodynamic analyses of reader–H3K4me3 and reader–H3K_C_4me3 binding, we carried out molecular dynamics (MD) simulations to provide an insight on flexibility of these five readers when complexed with H3K_C_4me3 and H3K4me3 (Figure 3). Starting structures were built by manually replacing the Kme3 residue of H3K4me3 with K_C_me3 in crystal structures of reader proteins, solvated in a 10 Å truncated octahedral box of TIP3P water [26], and neutralized explicitly with either sodium or chloride ions. AMBER12 [27] was then used to simulate the systems for 10 ns each, as previously described [11].

Molecular mechanics-generalized born surface area (MM-GBSA) binding free energy calculations were performed at 500 ps intervals over 10 ns to examine effects on electrostatically dominated cation–π interactions among the systems (Figure 3B, Table S1). The electrostatic contribution (Δ*E*_ele_) to the binding free energy was found to be slightly more favorable for the Kme3 compared to K_C_me3 for all systems except BPTF_PHD_ and TAF3_PHD_. For BPTF_PHD_, this can be explained by an unexpected stabilization of the K_C_me3 terminal ammonium group ~10 Å away from the π-face of W32. Interestingly, the positively charged residue was instead observed interacting with the negatively charged Glu19 residue side chain located on a flexible loop region of BPTF_PHD_ (Figure 3A). After 5 ns, K_C_me3 formed the interaction with Glu19 for the remainder of the simulation, unlike Kme3, shown by a distance vs. time plot of the N_ε_^+^ atom distance of both methylated lysines to the Glu19 carboxy group (Figure 3C). In the case of TAF3_PHD_, similar Δ*E*_ele_ values for K_C_me3 and Kme3 were likely a result of a closer interaction of K_C_me3 with W868-W891, rather than a residue located externally from the aromatic cage (Figure S5). A high degree of flexibility observed by the H3 backbone atoms in the simulation with TAF3_PHD_, but could have facilitated prioritization of the cation–π interaction.

The binding poses of residues Kme3 and K_C_me3 when complexed with all five reader proteins are shown at times 0, 5 and 10 ns, with corresponding distance vs. time plots of each N_ε_^+^ to the centroid of the aromatic cage residue side chains (Figures S2–S6 and Tables S2–S3). All K_C_me3 complexes maintained a cation–π interaction for a significant part of the simulation time with each of the aromatic cage residues, based on an established geometric cut-off of 6 Å [28], apart from BPTF_PHD_, and SGF29_TTD_ residue F264, for which neither K_C_me3 nor Kme3 formed this interaction (Figure S6). Overall, the binding poses were extremely similar, likely due to the structural similarities of the K_C_me3 and Kme3 trimethylammonium placement in the aromatic cages.

Next, we have quantum-chemically analyzed the energetics and bonding mechanism of TRP2 (a model for the two tryptophan residues of KDM5A_TTD_) with K_C_me3, and for comparison, Kme3, using dispersion-corrected density functional theory at BLYP-D3BJ/TZ2P and COSMO for simulating the aqueous solution [29]. Our model complexes cover those moieties of the KDM5A–H3K4me3 X-ray structure that contribute to the intermolecular interaction in the full reader–histone complexes. TRP2–Kme3 was terminated with one hydrogen at C_β_ of the Kme3 side chain and one hydrogen at each C_β_ of the TRP2 fragment. For K_C_me3, we have used the same X-ray structure as for Kme3, but, with one CH_2_ substituted by an S atom. To simulate the structural rigidity that is imposed by the protein backbone in the full protein system, the TRP2 fragment was kept frozen to the X-ray structure, both as a separate fragment and in the complexes. The Kme3 fragment was fully optimized, not only as the isolated molecule but also as the molecular fragment in its complex with TRP2. The K_C_me3 fragment was also fully optimized as the isolated molecule; but in the complex with TRP2, the carbon of the α-methyl group was kept constrained at the same position with respect to TRP2 as the α-methyl group in the TRP2–Kme3. The latter constraint simulates that, in the real complex, the remainder of K_C_me3, which is not present in our simple model, is kept at its position relative to KDM5A_TTD_ through intermolecular interactions in the same way as Kme3. The geometry of the optimized Kme3 model system differs only very slightly from the X-ray structure.

The new TRP2–K_C_me3 model complex presents a similar, although 2.1 kcal mol^−1^ weaker, bonding interaction than TRP2–Kme3: ∆*E*(aq) = −8.1 and −10.2 kcal mol^−1^ for TRP2–K_C_me3 and TRP2–Kme3 complexes, respectively (Table 2). This finding supports experimental observations and MD simulations that assign a quite comparable binding affinity for both of them. The geometries of the two model systems are similar, with NMe_3_^+^ in TRP2–Kme3 a bit closer to the TRP2 tryptophan cage than that in TRP2–K_C_me3. The shortest H•••C distance between a H atom of the K_C_me3 NMe_3_^+^ group and a C atom of the tryptophan (in the 5-membered ring) of the TRP2–K_C_me3 model is 2.88 Å, which has to be compared with the corresponding shortest H•••C distance of 2.78 Å in the TRP2–Kme3 complex (Table 2 and Figure S7). Likewise, another H atom of the same methyl group yields the shortest H•••C distance to the other TRP unit (6-membered ring), and this distance is also slightly longer in TRP2–K_C_me3 (2.94 Å) compared to 2.88 Å for TRP2–Kme3. Thus, the slightly longer intermolecular H•••C distances reflect the slightly weaker interaction in TRP2–K_C_me3 as compared to TRP2–Kme3.

Interestingly, our bonding analyses reveal that the instantaneous interaction energies ∆*E*_int_(aq) are in reverse order as compared to the net bond energies, although differences are relatively small: ∆*E*_int_(aq) amounts to −10.7 and −10.3 kcal mol^−1^ for TRP2–K_C_me3 and TRP2–Kme3, respectively (Table 2). This order in instantaneous interaction energies is inverted by the slightly more destabilizing strain energy ∆*E*_strain_(aq) associated with geometrical deformations of K_C_me3 in TRP2–K_C_me3. Thus, while this strain is negligible (only 0.1 kcal mol^–1^) for TRP2–Kme3, it becomes 2.6 kcal mol^–1^ in the case of TRP2–K_C_me3. The larger ∆*E*_strain_(aq) in the latter case is a consequence of the position of the S atom, which escapes from the linearity of the zig-zag shape of the K_C_me3 system when it interacts with TRP2, at variance with Kme3 (Figure S8). When K_C_me3 is allowed to fully relax, without TRP2, the same linearity as for Kme3 is achieved. For comparison, the CCCC dihedral angle in Kme3 in the complex is 177.6°, whereas the equivalent CCSC dihedral in K_C_me3 is 168.2°. This deviation is responsible for the larger strain energy in TRP2–K_C_me3 and the corresponding weaker ∆*E*_int_(aq) discussed above. This deviation also occurs if K_C_me3 is allowed to fully relax, i.e., without frozen C_α_ position. The interaction energy ∆*E*_int_ between the exact same structures but in the absence of aqueous solvation is again in favor of TRP–K_C_me3, namely, by 1.9 kcal mol^−1^. The desolvation incurred upon binding is 1.5 kcal mol^−1^ more destabilizing for the K_C_me3 than for the Kme3 complex, because of the presence of a sulfur atom, instead of CH_2_ group, in the former.

**Figure 4:**
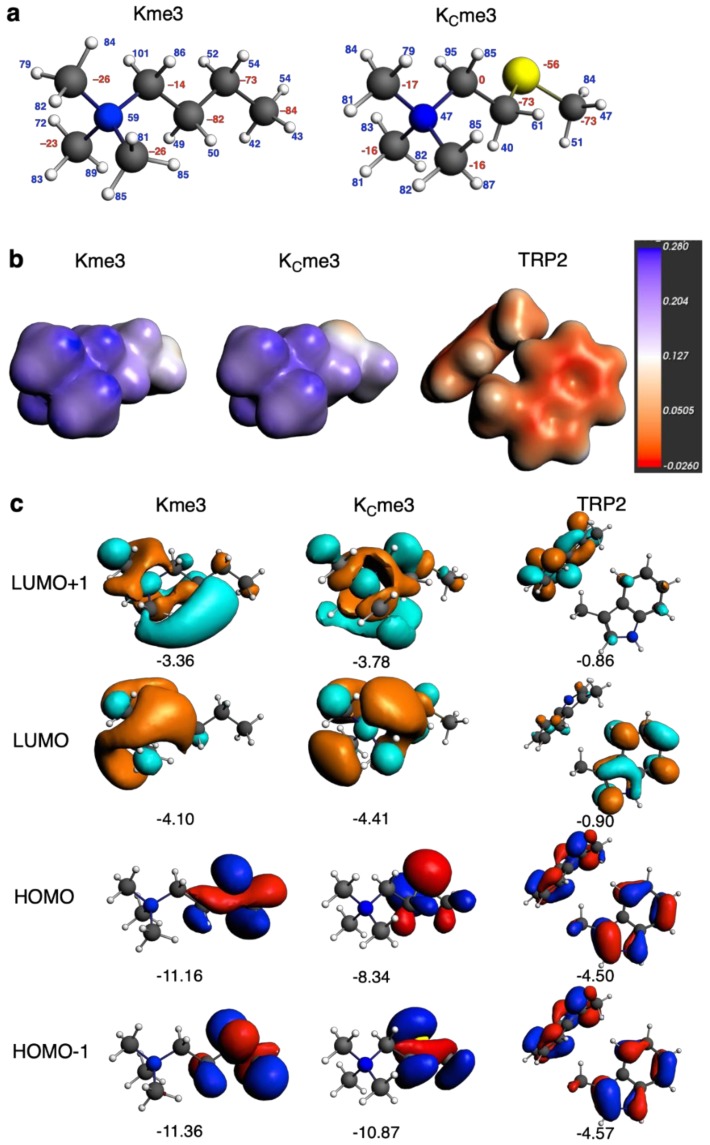
Computational analysis of Kme3, K_C_me3 and TRP2, computed at BLYP-D3BJ/TZ2P using frozen on X-ray structures for TRP2 and positions of C_α_ and full geometrical relaxation for all other portions: (**a**) Voronoi Deformation Density (VDD) atomic charges (in milli-a.u.; red = negative, blue = positive); (**b**) molecular electrostatic potential isosurfaces for Kme3, K_C_me3 and TRP2 (in a.u.); (**c**) frontier orbitals (isosurafce at 0.03) and orbital energies (in eV).

The more stabilizing intrinsic interaction energy of TRP2 with K_C_me3 (∆*E*_int_ = –29.5 kcal mol^–1^) than with Kme3 (∆*E*_int_ = –27.6 kcal mol^–1^) is further analyzed using quantitative Kohn–Sham molecular orbital (KS-MO) and an associated canonical energy decomposition analysis (EDA); see Table 2. This analysis reveals more favorable electrostatic, orbital and dispersion interactions as the origin of the stronger interaction ∆*E*_int_ term in TRP2–K_C_me3. The more attractive electrostatic interaction ∆*V*_elstat_ is due to the presence of the negatively charged sulfur atom, which comes in close proximity to the positively charged H atoms of one of the TRP units (see the Voronoi Deformation Density (VDD) charges in Figure 4A). This is also supported by the molecular electrostatic potential isosurfaces for Kme3, K_C_me3 and TRP2 (Figure 4B) [30,31]. The S atom in K_C_me3 appears redder (more towards negative), whereas its trimethylated group is bluer (more positive) than for Kme3, which favors the interaction of the former with TRP2.

Likewise, a favorable spatial configuration also enhances the attractive ∆*E*_oi_ term in TRP2–K_C_me3 through a larger orbital overlap and thus more stabilizing donor–acceptor orbital interactions between occupied TRP2 π orbitals and empty acceptor σ*_C-H_ type orbitals on the K_C_me3 side chain (Figure 4C; three out of the four overlaps between the frontier FMOs are larger for TRP2–K_C_me3, as can be seen in Table S5) [32,33]. The torsion of the zig-zag chain caused by the S atom makes the three central members of the side chain of K_C_me3 (S–CH_2_-CH_2_) to be closer to one of the TRP2 units in TRP2–K_C_me3 than the equivalent CH_2_–CH_2_–CH_2_ side chain for Kme3 in TRP2–Kme3. This translates into a more destabilizing closed-shell–closed-shell Pauli repulsion ∆*E*_Pauli_ in the TRP2–K_C_me3 complex. Taken together, the TRP2–K_C_me3 complex differs only slightly in stability from the TRP2–Kme3 complex. The former appears to be only slightly less stable due to somewhat more pronounced structural deformations reflected by ∆*E*_strain_(aq) and a somewhat more endothermic desolvation Δ*E*_int_(desolv) (Table 2).

## 3. Discussion

To assess whether trimethylthialysine can serve as an easily accessible and functional analogue of trimethyllysine when studying molecular recognition processes in epigenetics, we have carried out comparative thermodynamic analyses, molecular dynamics simulations and quantum chemical analyses for binding of epigenetic reader proteins with histone peptides bearing trimethyllysine and trimethylthialysine. ITC data showed that H3K4me3 and H3K_C_4me3 exhibit similar binding affinities for a panel of five human reader proteins. These observations were further supported by molecular dynamics simulations that demonstrated that the binding poses for the two residues were very similar, and by quantum chemical analyses that showed that similar bonding interactions are present for Kme3 and K_C_me3 with TRP2. The study further establishes trimethylthialysine as a widely applicable trimethyllysine mimic, which can be used to study genuinely important biomolecular processes that involve the methylation of lysine in a more precise fashion, in vitro and in the cellular environment, thereby helping to unravel the complex language of post-translational modifications and its role on the nucleosome and higher order chromatin structures in biology.

## 4. Materials and Methods

### 4.1. Preparation of H3K4me3 and H3K_C_4me3

H3K4me3 and H3C4 were synthesized by solid-phase peptide synthesis (Schemes S1 and S2). Cysteine was alkylated to produce trimethylated lysine analogue (K_C_me3) as described previously [22]. The reaction was performed using thermomixer (Eppendorf Thermomixer R, Hamburg, Germany). For synthesizing the 1–10 H3K_C_4me3: 50 mg of purified and lyophilized unalkylated peptide was dissolved in 4.9 mL alkylation buffer (4 M GuHCl, 1 M HEPES pH 7.8 and 10 mM d/l-methionine) and allowed to incubate for 1 h at 37 °C under reducing conditions by adding 100 µL 1 M DTT. (2-Bromoethyl) trimethylammonium bromide was directly dissolved into the reaction mixture and allowed to react at 50 °C. After 2.5 h reaction time, 10 µL 1 M DTT was added to the reaction mixture and the reaction was allowed to proceed for another 2.5 h. The reaction was quenched by incubating the reaction mixture with 25 µL of 2-mercaptoethanol for 30 min at room temperature, and then directed to freeze-dryer overnight followed by prep-HPLC purification (Figures S9–S11).

### 4.2. Reader Domain expression and Purification

The methylated reader proteins were expressed and purified as previously described [29].

### 4.3. Isothermal Titration Calorimetry

Reader proteins used for ITC experiments were produced as described [9]. ITC experiments were carried out at 298.15 K using a fully automated MicroCal Auto-iTC200 (GE Healthcare, Northampton, MA, USA). Histone peptides and reader proteins were dissolved in the same buffer (used in size exclusion chromatography). Each ITC titration consisted of 19 injections of histone peptide (0.3–1.2 mM) to reader protein (22–100 µM). Experiments were repeated 3 to 5 times. Heats of dilution for histone peptides were determined in control experiments, and were subtracted from the titration binding data before curve fitting. Curve fitting was performed using Origin 6.0 (Microcal Inc., Northampton, MA, USA) with a one-site note.

### 4.4. Molecular Dynamics Simulations

Ten MD simulations were carried out for 10 ns each. PDB structures for the models representing TAF3_PHD_ (PDB: 2K17), KDM4A_TTD_ (PDB: 2GFA), KDM5A_PHD3_ (PDB: 2KGI), BPTF_PHD_ (PDB: 2F6J), and SGF29_TTD_ (PDB: 3ME9) reader proteins were used as a template for building the reader bound to K_C_me_3_ and Kme3 systems.

Hydrogen atom addition was performed with *tLeap* [34]. Systems were solvated in a truncated octahedral box of TIP3P [25] that extended at least 10 Å from protein atoms and neutralized explicitly with either Na^+^ or Cl^−^ counterions.

AMBER12 (San Francisco, CA, USA, 2012) [26,27] was used with the Amberff12SB force field to define protein partial charges. The force constants for bond, angle, and torsions for the atoms bonded to zinc derived from the Zinc AMBER Force Field (ZAFF) developed by the Merz group [35]. Atomic partial charges for each atomic center in K_C_me_3_ correspond to those derived using the Restrained Electrostatic Potential (RESP) [36] module in AmberTools [37] calculated using HF/6–31G(d), shown in Table S3. Parameters for Kme3 were previously derived also using the RESP methodology [11].

The ten systems were then minimized in two steps. First came 1000 steps of steep descent and 1000 steps of conjugate gradient minimization wherein the protein was held fixed by using position restraints with a force constant of 500 kcal mol^−1^ Å^−2^. This was repeated without the position restraints. The system was then heated for 1 ns from 0 to 310 K under constant volume periodic boundary conditions (NVT). Then, 1 ns of equilibration under constant pressure and temperature (NPT) was performed. Following this, 10 ns molecular dynamics simulations were then performed.

Langevin thermostat [38] was used to simulate a constant temperature of 310 K with collision frequency of 1 ps^−1^. The SHAKE algorithm [39] was turned on to constrain all bonds involving hydrogen and 2 fs was defined as the time step for numerical integration. Isotropic position scaling was used to maintain the pressure of 1 atm (τ*_p_* = 2 ps). The particle mesh Ewald summation method [40] was employed to enforce an 8.0 Å cutoff for non-bonded long-range and electrostatic interactions.

Trajectories were visualized using visual molecular dynamics (VMD 1.9.2., Champaign, IL, USA) [41]. Endstate free energies and electrostatic energies were calculated using MM-GBSA calculations [42]. Energy values were measured every 500 ps over 10 ns. A salt concentration of 0.15 M was used to parallel physiological conditions. For cation distance calculations, the π-system was defined for aromatic cage residues as the centroid of the side chain aromatic (non-H) atoms.

### 4.5. Quantum Chemical Analysis

All calculations were carried out with the Amsterdam Density Functional (ADF 2018, Amsterdam, The Netherlands)) program using dispersion-corrected density functional theory at the BLYP-D3BJ/TZ2P level of theory (Table S5) [43]. The effect of aqueous solvation was simulated by means of the conductor like screening model (COSMO) of solvation, as implemented in ADF. The approach has been benchmarked against highly correlated post-Hartree–Fock methods and experimental data and was found to work reliably [44,45,46,47,48,49].

The bonding mechanism in our model complexes have been further analyzed using quantitative (Kohn–Sham) molecular orbital (MO) theory in combination with an energy decomposition analysis (EDA) [50,51,52]. The bond energy in aqueous solution ∆*E*(aq) consists of two major components; namely, the strain energy ∆*E*_strain_(aq) associated with deforming the Kme3 and the reader from their own equilibrium structure to the geometry they adopt in the complex, plus the interaction energy ∆*E*_int_(aq) between these deformed solutes in the complex (see Equation (1)):

∆*E*(aq) = ∆*E*_strain_(aq) + ∆*E*_int_(aq). (1)

To arrive at an understanding of the importance of desolvation phenomena during the complexation process, we separate the solute–solute interaction ∆*E*_int_(aq) into the effect caused by the change in solvation ∆*E*_int_(desolv) and the remaining intrinsic interaction ∆*E*_int_ between the unsolvated fragments in vacuum ∆*E*_int_:

∆*E*_int_(aq) = ∆*E*_int_(desolv) + ∆*E*_int_. (2)

In the EDA, the intrinsic interaction energy Δ*E*_int_ can be further decomposed, as shown in Equation (3):

∆*E*_int_ = ∆*V*_elstat_ + ∆*E*_Pauli_ + ∆*E*_oi_ + ∆*E*_disp_. (3)

Here, ∆*V*_elstat_ corresponds to the classical electrostatic interaction between the unperturbed charge distributions of the deformed fragments, which is usually attractive. The Pauli repulsion ∆*E*_Pauli_ comprises the destabilizing interactions between occupied orbitals and is responsible for the steric repulsions. The orbital interaction ∆*E*_oi_ accounts for charge transfer (donor–acceptor interactions between occupied orbitals on one moiety with unoccupied orbitals of the other, including the HOMO–LUMO interactions) and polarization (empty/occupied orbital mixing on one fragment due to the presence of another fragment). Finally, the ∆*E*_disp_ term accounts for the dispersion interactions based on Grimme′s DFT-D3BJ correction. Furthermore, the charge distribution has been analyzed using the Voronoi deformation density (VDD) method [53].

## Figures and Tables

**Table 1:** Thermodynamic parameters for association of the 10-mer H3K4me3 and H3K_C_4me3 peptides (ART(Kme3/K_C_me3)QTARKS) with epigenetic reader proteins (values and errors were obtained from 3–5 repeated ITC experiments carried out at 298.15 K).

	H3K4me3				H3K_C_4me3			
	*K*_d_ (μM)	Δ*G*° (kcal mol^−1^)	Δ*H*° (kcal mol^−1^)	−TΔ*S*° (kcal mol^−1^)	*K*_d_ (μM)	Δ*G*° (kcal mol^−1^)	Δ*H*° (kcal mol^−1^)	−TΔ*S*° (kcal mol^−1^)
KDM5A_PHD_	0.071 ± 0.008	−9.7 ± 0.1	−10.7 ± 0.1	1.0 ± 0.1	0.15 ± 0.2	−9.3 ± 0.1	−9.6 ± 0.1	0.3 ± 0.1
TAF3_PHD_	0.084 ± 0.012	−9.6 ± 0.1	−10.7 ± 0.1	1.1 ± 0.1	0.042 ± 0.007	−10.1 ± 0.1	−10.8 ± 0.1	0.7 ± 0.1
BPTF_PHD_	1.9 ± 0.2	−7.8 ± 0.1	−12.4 ± 0.1	4.6 ± 0.1	3.8 ± 0.5	−7.4 ± 0.1	−9.0 ± 0.1	1.6 ± 0.1
SGF29_TTD_	2.6 ± 0.3	−7.6 ± 0.1	−8.0 ± 0.1	0.4 ± 0.1	6.1 ± 0.7	−7.1 ± 0.1	−5.8 ± 0.1	−1.3 ± 0.2
KDM4A_TTD_	6.6 ± 0.8	−7.1 ± 0.1	−13.0 ± 0.2	5.9 ± 0.2	3.1 ± 0.6	−7.5 ± 0.1	−14.8 ± 0.2	7.3 ± 0.2

**Table 2:** Quantum-chemical bonding analysis (energies in kcal mol^−1^, distances in Å) in TRP2–Kme3 and TRP2–K_C_me3 systems in aqueous solution. ^1^

	TRP2–Kme3 ^2^	TRP2–K_C_me3 ^3^
Δ*E*(aq)	−10.2	−8.1
Δ*E*_strain_(aq)	0.1	2.6
Δ*E*_int_(aq)	−10.3	−10.7
Δ*E*_int_(desolv)	17.3	18.8
Δ*E*_int_	−27.6	−29.5
Δ*E*_Pauli_	20.8	24.3
Δ*V*_elstat_	−15.0	−17.0
Δ*E*_oi_	−13.0	−14.4
Δ*E*_disp_	−20.4	−22.3
d(H_Me_-C_TRP−6MR_)	2.88	2.94
d(H_Me_-C_TRP−5MR_)	2.78	2.88

^1^ Computed at BLYP-D3BJ/TZ2P with COSMO to simulate aqueous solution. Structural rigidity imposed by the protein backbone is simulated through constrained geometry optimizations. See also Equations (1)–(3) in the Experimental section. ^2^ TRP2 frozen, Kme3 entirely free. ^3^ TRP2 frozen, α-methyl carbon fixed to position TRP2–Kme3 optimization.

## Supplementary Materials

The following are available online. Scheme 1: Solid-phase synthesis of histone peptide H3K4me3. Figure S1: ITC data. Figure S2: MD simulations of BPTF_PHD_. Figure S3: MD simulations of KDM4A_TTD_. Figure S4: MD simulations of KDM5A_PHD3_. Figure S5: MD simulations of TAF3_PHD_. Figure S6: MD simulations of SGF29_TTD_. Figure S7: Top view of structure of TRP2–Kme3 and TRP–K_C_me3 model complexes. Figure S8: Front view of the structure of TRP–Kme3 and TRP2–K_C_me3 model complexes. Figure S9: LC-MS analysis of 1–10 H3C4 after RP-HPLC purification. Figure S10: LC-MS analysis of 1–10 H3K_C_4me3 after RP-HPLC purification. Figure S11: LC-MS analysis of 1–10 H3K4me3 after RP-HPLC purification. Table S1 Concentrations of protein and peptide, with C-value and N binding cites in ITC binding studies. Table S2: MM-GBSA binding free energies and electrostatic contributions calculated for Kme3 and K_C_me3 complexed with reader proteins over 10 ns at 500 ps intervals. Table S3: Average root mean square deviation (RMSD) and error of C_α_ atoms of reader proteins. Table S4: Cartesian coordinates and charges calculated using the RESP method HF/6–31G* of modified K_C_me3. Table S5: Overlaps between the MOs of TRP and Kme3 or K_C_me3. Table S6: Cartesian coordinates (in Å) of TRP2–Kme3 and TRP2–K_C_me3complexes, computed at BLYP-D3BJ/TZ2P using COSMO to simulate aqueous solvation and a constrained optimization to simulate the effect of the protein backbone.
