# Supplementary material for: Comparison of Molecular Recognition of Trimethyllysine and Trimethylthialysine by Epigenetic Reader Proteins
Source: Molecules. 2020 Apr 21;25(8):1918. doi: 10.3390/molecules25081918 (PMC7221964; doi:10.3390/molecules25081918)
Supplement: Supplementary file 1 [file molecules-25-01918-s001.pdf]

Supporting Information

Comparison of Molecular Recognition of Trimethyllysine and Trimethylthialysine by Epigenetic Reader Proteins

Jordi C. J. Hintzen [1,†], Jordi Poater [2,†], Kiran Kumar [3,†], Abbas H. K. Al Temimi [4,†], Bas J. G. E. Pieters [4], Robert S. Paton [3,*], F. Matthias Bickelhaupt [4,5,*] and Jasmin Mecinović [1,4,*]

[1] Department of Physics, Chemistry and Pharmacy, University of Southern Denmark, Campusvej 55, 5230 Odense, Denmark

[2] ICREA and Departament de Química Inorgànica i Orgànica & IQTCUB, Universitat de Barcelona, Martí I Franquès 1–11, 08028 Barcelona, Spain

[3] Chemistry Research Laboratory, University of Oxford, 12 Mansfield Road, Oxford OX1 3TA, UK

[4] Institute for Molecules and Materials, Radboud University, Heyendaalseweg 135, 6522 AJ Nijmegen, Netherlands

[5] Department of Theoretical Chemistry and Amsterdam Center for Multiscale Modeling, Vrije Universiteit Amsterdam, De Boelelaan 1083, 1081HV Amsterdam, Netherlands

* Correspondence: robert.paton@chem.ox.ac.uk (R.S.P.); f.m.bickelhaupt@vu.nl (F.M.B.); mecinovic@sdu.dk (J.M.)

† These authors contributed equally to this work.

Table of Contents

1. **General Information** 3
2. **Synthesis and Purification of Histone Peptides and Reader Proteins** 3
3. **ITC Measurements** 6
4. **Molecular Dynamics Simulations** 8
5. **Quantum Chemical Analysis** 16
6. **LC-MS of Purified Histone Peptides** 21

1. General Information

1.1. Methods

High resolution masses were recorded with a JEOL AccuTOF CS JMS-T100CS mass spectrometer. LS-MS analysis for all the compounds was performed on a Thermo Finnigan LCQ-Fleet ESI-ion trap (Thermofischer, Breda, the Netherlands) equipped with a Phenomenex Gemini-NX C18 column, 50 × 2.0 mm, particle size 3 μM (Phenomenex, Utrecht, the Netherlands). An acetonitrile/water gradient containing 0.1% formic acid was used for elution (5%–100%, 1–50 min, flow 0.2 mL min-1). The room temperature in the reactions is in the range 20–25 °C. Lyophilization was achieved using an ilShin Freeze Dryer (ilShin, Ede, the Netherlands).

1.2. Materials

All reagents were obtained from commercial sources and used without further purifications. Fmoc amino acid derivatives, *N,N'*-Diisopropylcarbodiimide (DIC) and 1-Hydroxybenzotriazole (HOBt) were obtained from Novabiochem (EMD Chemicals, Gibbstown, USA). Triisopropylsilane (TIPS), *N'N*-diisopropylethylamine (DIPEA), trifluoroacetic acid (TFA), (2-Bromoethyl)trimethylammonium bromide and piperidine were purchased from Sigma-Aldrich. *N,N*-dimethylformamide (DMF) solvent for peptide synthesis and gradient degree high-performance liquid chromatography (HPLC) acetonitrile were purchased from Actu-All Chemicals b.v. (Oss, the Netherlands).

2. Synthesis and Purification of Histone Peptides and Reader Proteins

2.1. Synthesis of Histone Peptides

The general synthesis strategy of 10-mer natural histone peptide is outlined in Scheme S1.

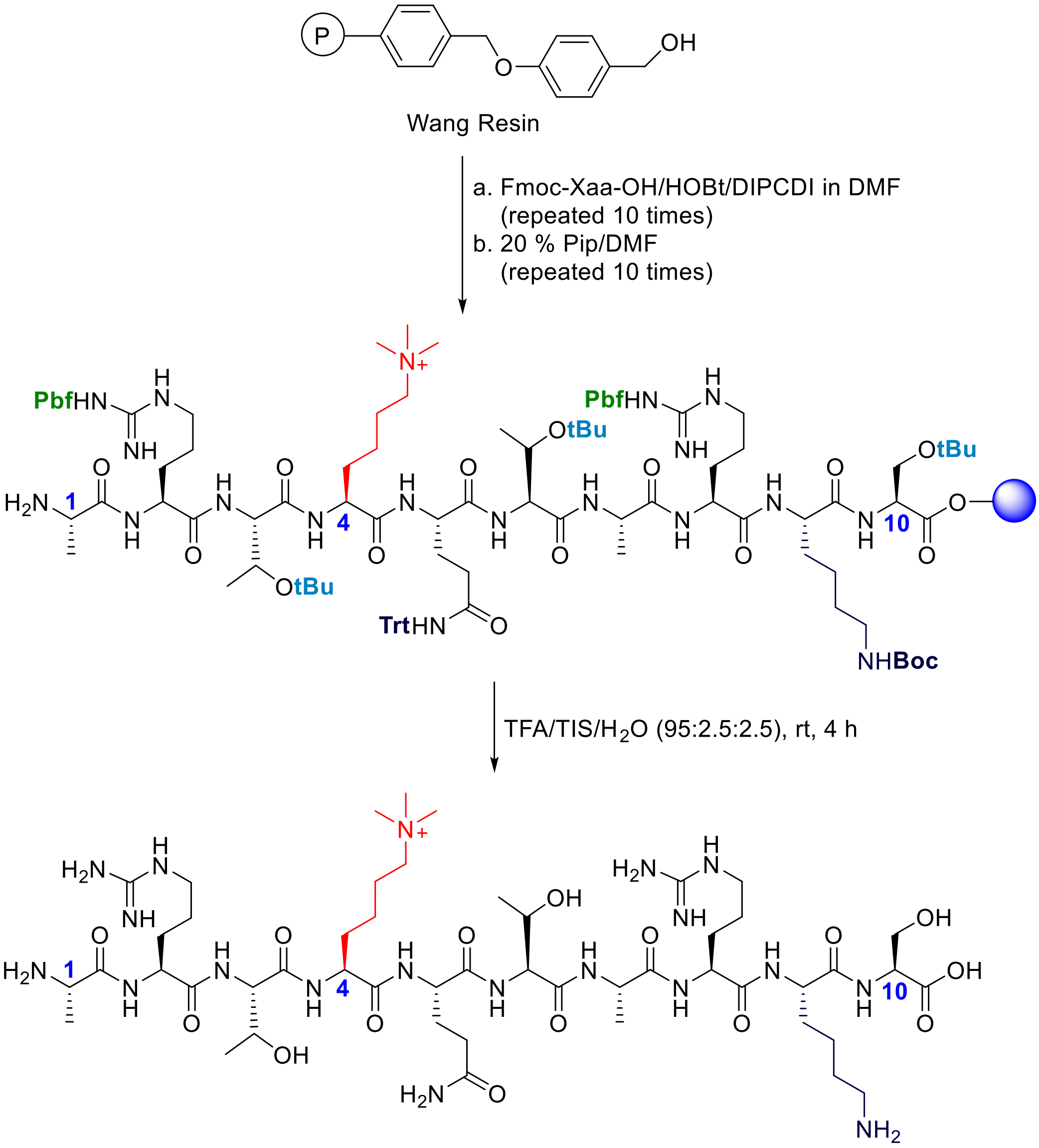

Scheme S1. Solid-phase synthesis of histone peptide H3K4me3.

2.2 Analytical HPLC of Histone Peptides

Lyophilized crude peptides were purified by prep-HPLC on a Phenomenex® Gemini-NX 3u C18 110A reversed-phase column (150 × 21.2 mm) using gradient elution at constant flow rate of 10 mL/min and the temperature is 30 °C. A typical run was performed as follows:

For 1-10 H3K$_{C}$4me3; after 3 mins at 2% a gradient of 2% to 10% over 10 mins was introduced, followed by a gradient of 10% to 100% over 20 mins and from 100% to 100% over 25 mins finalized by 5 mins at 100% CH3CN (total runtime 30 mins).

For 1-10 H3K$_{C}$4; after 3 mins at 3% a gradient of 3% to 15% over 12 mins was introduced, followed by a gradient of 15% to 30% over 17 mins and from 30% to 100% over 19 mins, continuing from 100% to 100% over 21 mins finalized by 3 mins at 100% CH_3CN (total runtime 30 mins).

For 1-10 H3K4me3; after 3 mins at 3% a gradient of 3% to 3% over 6 mins was introduced, followed by a gradient of 3% to 100% over 10 mins and from 100% to 100% over 13 mins finalized by 4 mins at 100% CH_3CN (total runtime 20 mins). Solvent A was 0.1% trifluoroacetic acid in H_2O, Solvent B was 0.1% trifluoroacetic acid in acetonitrile. The pure fractions containing product were combined and freeze-dried overnight to yield the peptides as white off solid.

3. ITC Measurements

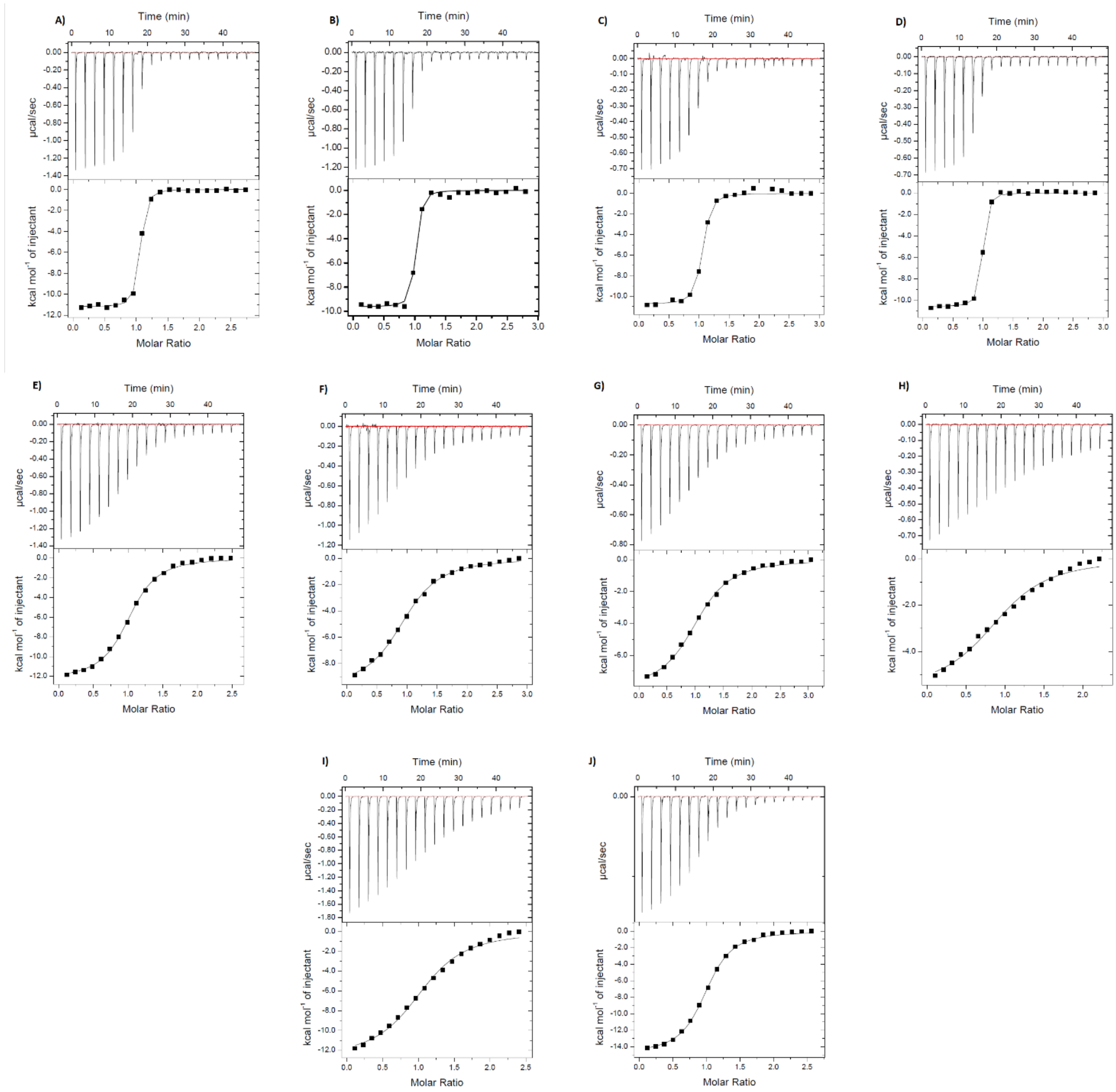

Figure S1. ITC data. Thermodynamic analyses showing binding of A) KDM5A$_{\text{PHD3}}$–H3K4me3; B) KDM5A$_{\text{PHD3}}$–H3K$_{\text{C}}$4me3; C) TAF3$_{\text{PHD}}$–H3K4me3; D) TAF3$_{\text{PHD}}$–H3K$_{\text{C}}$4me3; E) BPTF$_{\text{PHD}}$–H3K4me3; F) BPTF$_{\text{PHD}}$–H3K$_{\text{C}}$4me3; G) SGF29$_{\text{TTD}}$–H3K4me3; H) SGF29$_{\text{TTD}}$–H3K$_{\text{C}}$4me3; I) KDM4A$_{\text{TTD}}$–H3K4me3; J) KDM4A$_{\text{TTD}}$–H3K$_{\text{C}}$4me3.

Table S1. Concentrations of protein and peptide, with C-value and N binding cites in ITC binding studies.

	**H3K4me3**				**H3K$_c$4me3**			
	Protein conc. (μM)	Peptide conc. (μM)	C-value	N	Protein conc. (μM)	Peptide conc. (μM)	C-value	N
KDM5A$_{PHD}$	29	360	408	1.00–1.01	29	420	193	1.00–1.02
TAF3$_{PHD}$	20.5	300	244	1.00–1.01	20.5	300	488	0.98–1.02
BPTF$_{PHD}$	43	520	21.6	1.00–1.01	43	620	11.3	0.99–1.00
SGF29$_{TTD}$	30	490	11.5	1.00–1.02	51	550	8.4	0.98–1.00
KDM4A$_{TTD}$	58	680	13.3	0.98–1.01	100	1250	32.3	0.99–1.01

4. Molecular Dynamics Simulations

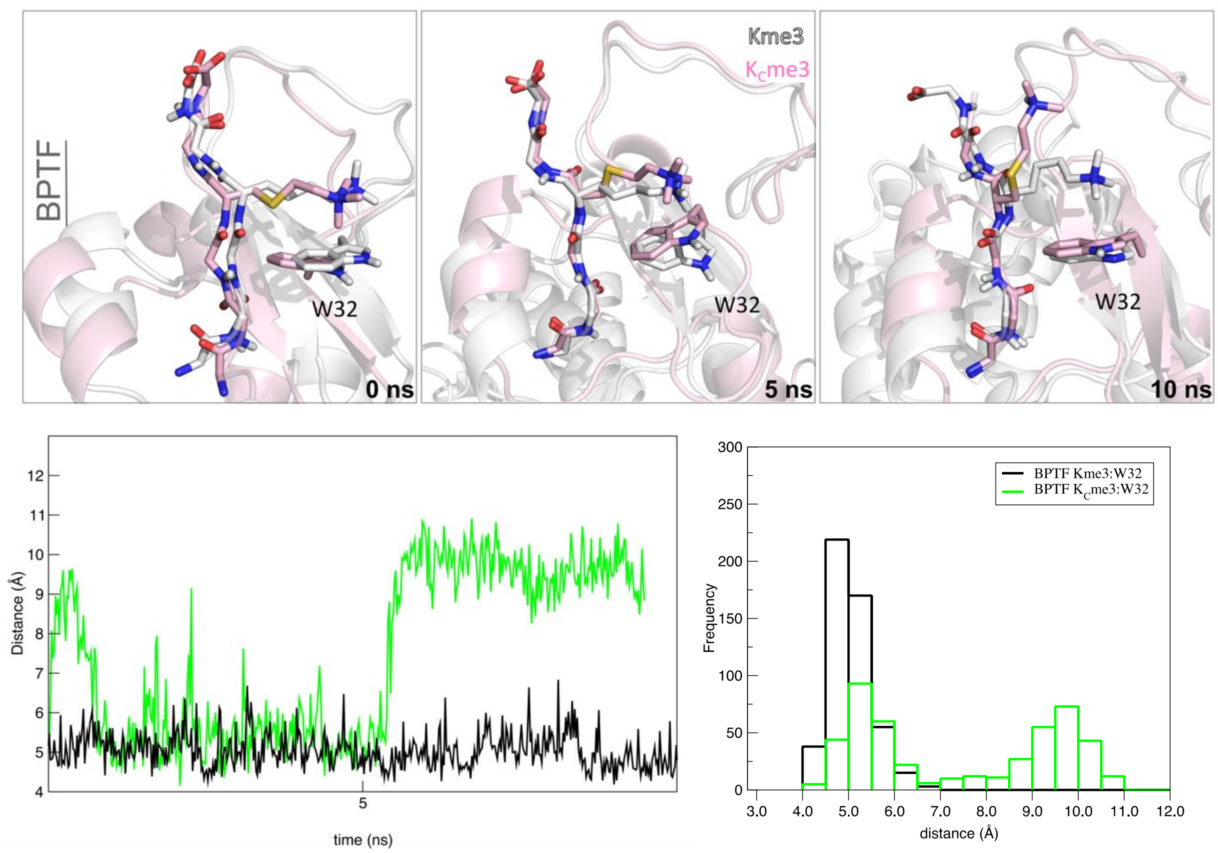

Figure S2. MD simulations of BPTFPHD. (Top) Snapshots of reader BPTFPHD complexed with histone 3 chain backbone (liquorice) containing KCme3 (pink) and Kme3 (white) active sites at times 0 ns, 5 ns and 10 ns. (Bottom) Distance vs. time plots of N^+ side chain atoms of Kme3 and KCme3 to W32 side chain center of mass over 10 ns.

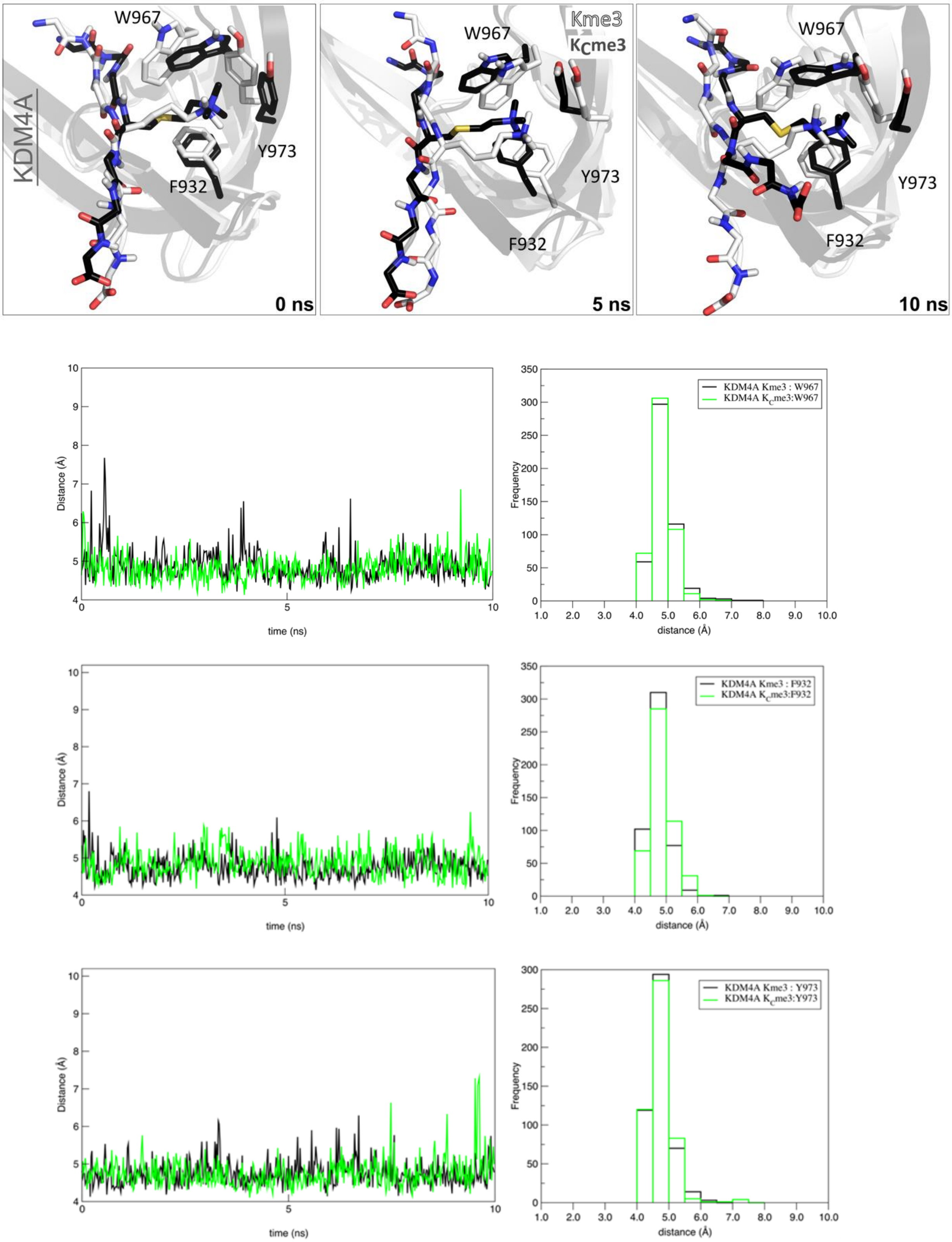

Figure S3. MD simulations of KDM4ATTD. (Top) Snapshots of reader KDM4ATTD complexed with histone 3 chain backbone (liquorice) containing KCme3 (black) and Kme3 (white) active sites at times 0 ns, 5 ns and 10 ns. (Bottom) Distance vs. time plots of N^+ side chain atoms of Kme3 and KCme3 to F932, W967 and Y973 side chain centers of mass over 10 ns.

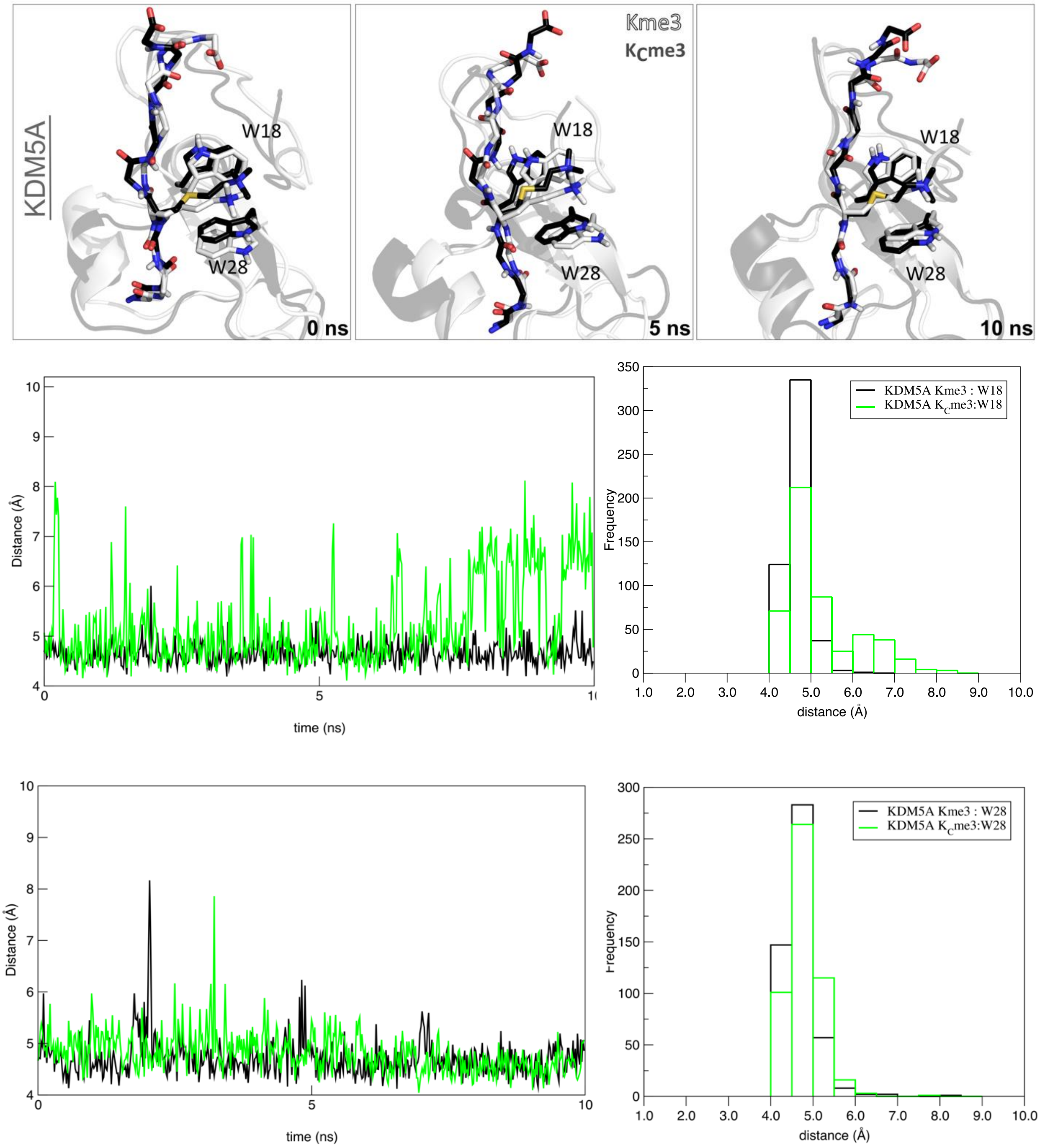

Figure S4. MD simulations of KDM5APHD3. (Top) Snapshots of reader KDM5APHD3 complexed with histone 3 chain backbone (liquorice) containing KCme3 (black) and Kme3 (white) active sites at times 0 ns, 5 ns and 10 ns. (Bottom) Distance vs. time plots of N^+ side chain atoms of Kme3 and KCme3 to W18 and W28 side chain centers of mass over 10 ns.

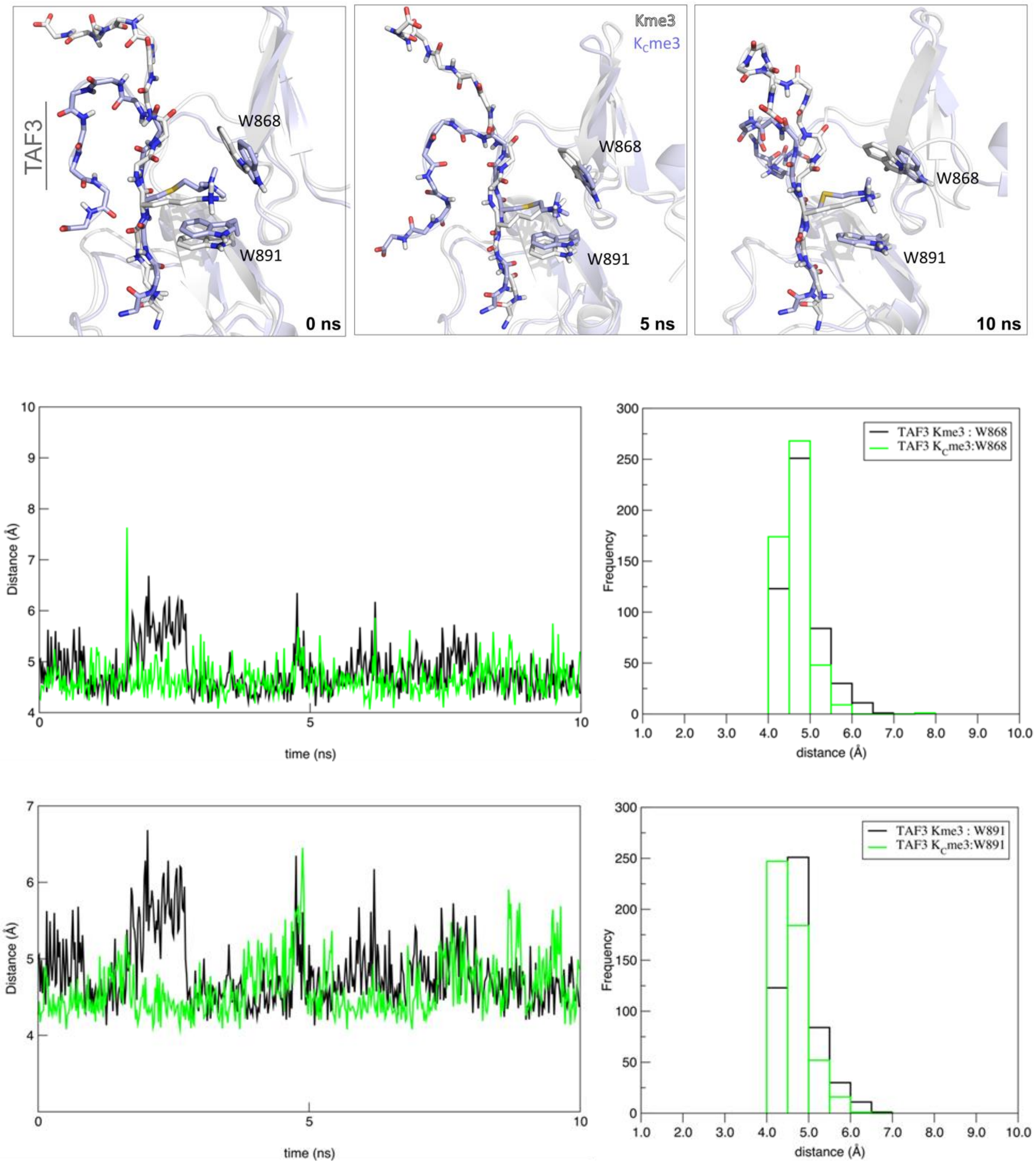

Figure S5. MD simulations of TAF3$_{PHD}$. (Top) Snapshots of reader TAF3$_{PHD}$ complexed with histone 3 chain backbone (liquorice) containing K$_C$me3 (blue) and Kme3 (white) active sites at times 0 ns, 5 ns and 10 ns. (Bottom) Distance vs. time plots of N$^+$ side chain atoms of Kme3 and K$_C$me3 to W868 and W891 side chain centers of mass over 10 ns.

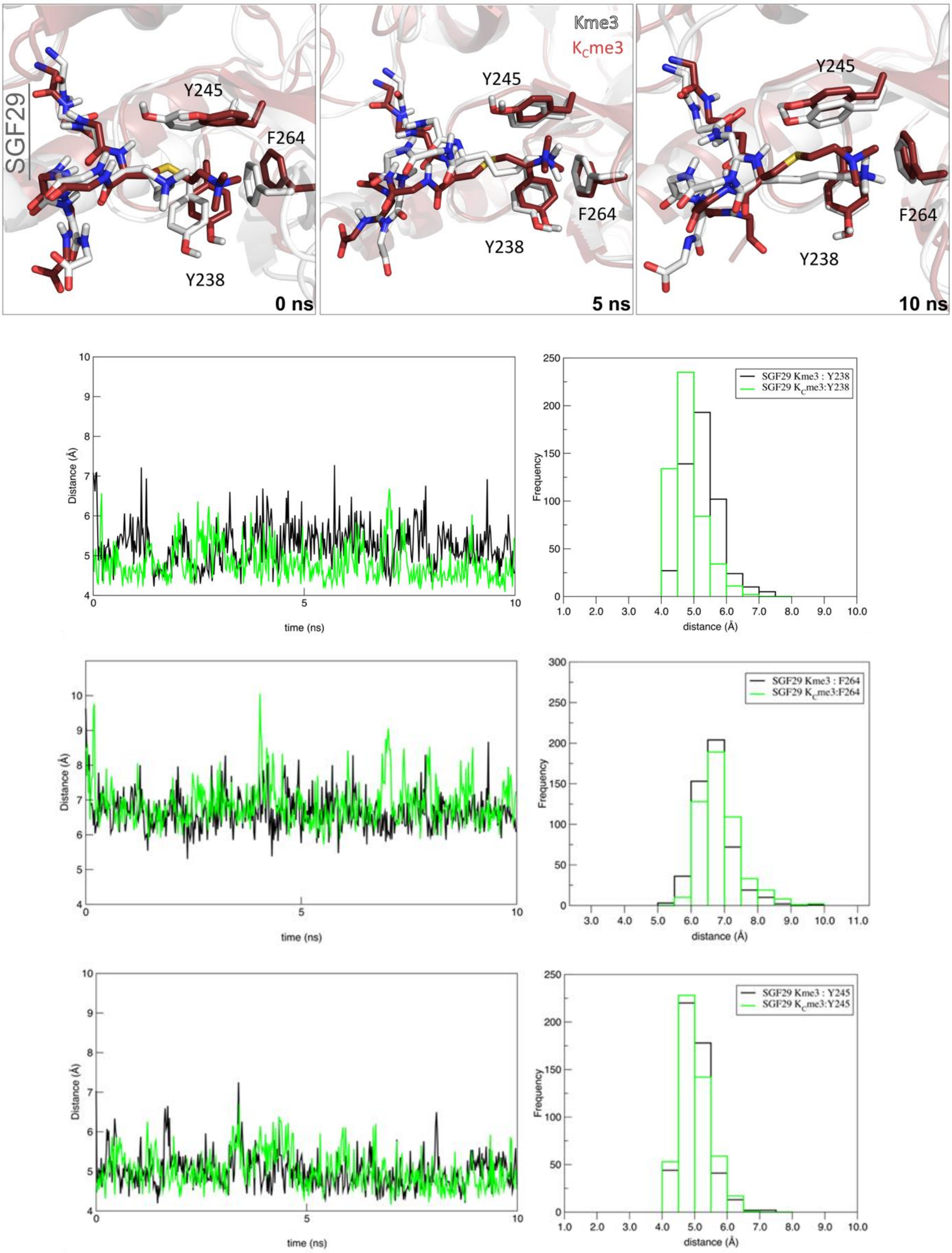

Figure S6. MD simulations of SGF29$_{TTD}$. (Top) Snapshots of reader SGF29$_{TTD}$ complexed with histone 3 chain backbone (liquorice) containing K$_C$me3 (red) and Kme3 (white) active sites at times 0 ns, 5 ns and 10 ns. (Bottom) Distance vs. time plots of N$^+$ side chain atoms of Kme3 and K$_C$me3 to F264, Y238 and Y245 side chain centers of mass over 10 ns.

Table S2. MM-GBSA binding free energies and electrostatic contributions calculated for Kme3 and Kcme3 complexed with reader proteins over 10 ns at 500 ps intervals.

System	MM-GBSA (kcal/mol)			
	**Kme3**		**Kcme3**	
	ΔG	ΔE_{ele}	ΔG	ΔE_{ele}
$BPTF_{PHD}$	–39.6	–194.8	–53.1	–239.3
$KDM4A_{TTD}$	–47.2	–283.5	–47.0	–266.0
$KDM5A_{PHD3}$	–42.4	–176.3	–41.8	–158.7
$SGF29_{TTD}$	–46.4	–197.7	–42.5	–177.8
$TAF3_{PHD}$	–45.5	–164.7	–44.3	–166.4

Table S3. Average root mean square deviation (RMSD) and error of C_{α} atoms of reader proteins.

System	RMSD (Å)			
	**Kme3**		**Kcme3**	
	Reader	H3	Reader	H3
$BPTF_{PHD}$	5.85 ± 2.49	0.47 ± 0.20	4.24 ± 1.67	0.79 ± 0.21
$KDM4A_{TTD}$	2.08 ± 0.64	1.17 ± 0.27	3.49 ± 0.68	0.75 ± 0.21
$KDM5A_{PHD3}$	2.45 ± 0.58	1.16 ± 0.34	2.06 ± 0.32	1.82 ± 0.51
$SGF29_{TTD}$	1.28 ± 0.20	1.03 ± 0.45	1.26 ± 0.14	0.97 ± 0.30
$TAF3_{PHD}$	3.25 ± 0.82	3.24 ± 1.20	2.53 ± 0.37	3.79 ± 1.65

Table S4. Cartesian coordinates and charges calculated using the RESP method HF/6-31G* of modified Kcme3.

Kcme3 Atom	X	Y	Z	RESP Charge
N	-3.531	1.376	0.222	-0.8584
C	-3.262	0.130	-0.448	0.5194
C	-1.933	-0.464	0.042	-0.3406
C	0.840	-0.328	-0.038	0.0041
C	2.062	0.585	-0.003	0.0217
N	3.412	-0.104	0.043	0.0771
C	-4.347	-0.927	-0.263	0.2879
O	-5.242	-0.803	0.503	-0.4361
C	3.644	-0.928	-1.182	-0.3320
C	4.460	0.964	0.105	-0.3320
C	3.539	-0.967	1.257	-0.3320
H	-3.882	1.209	1.147	0.3425
H	-4.237	1.896	-0.261	0.3425
H	-3.168	0.322	-1.513	0.0066
H	-1.679	-1.346	-0.536	0.1577
H	-2.011	-0.748	1.085	0.1577
H	0.863	-0.992	-0.892	0.0657
H	0.774	-0.923	0.864	0.0657
H	2.081	1.216	-0.880	0.1172
H	2.020	1.221	0.870	0.1172
H	-4.246	-1.831	-0.872	0.0297
H	3.514	-0.306	-2.055	0.1779
H	4.652	-1.314	-1.155	0.1779
H	2.946	-1.749	-1.206	0.1779
H	4.378	1.591	-0.770	0.1779
H	5.435	0.501	0.133	0.1779
H	4.310	1.556	0.995	0.1779
H	3.328	-0.375	2.135	0.1779
H	2.847	-1.790	1.191	0.1779
H	4.548	-1.349	1.308	0.1779
S	-0.608	0.772	-0.143	-0.2831

5. Quantum Chemical Analysis

5.1. Bonding Analysis

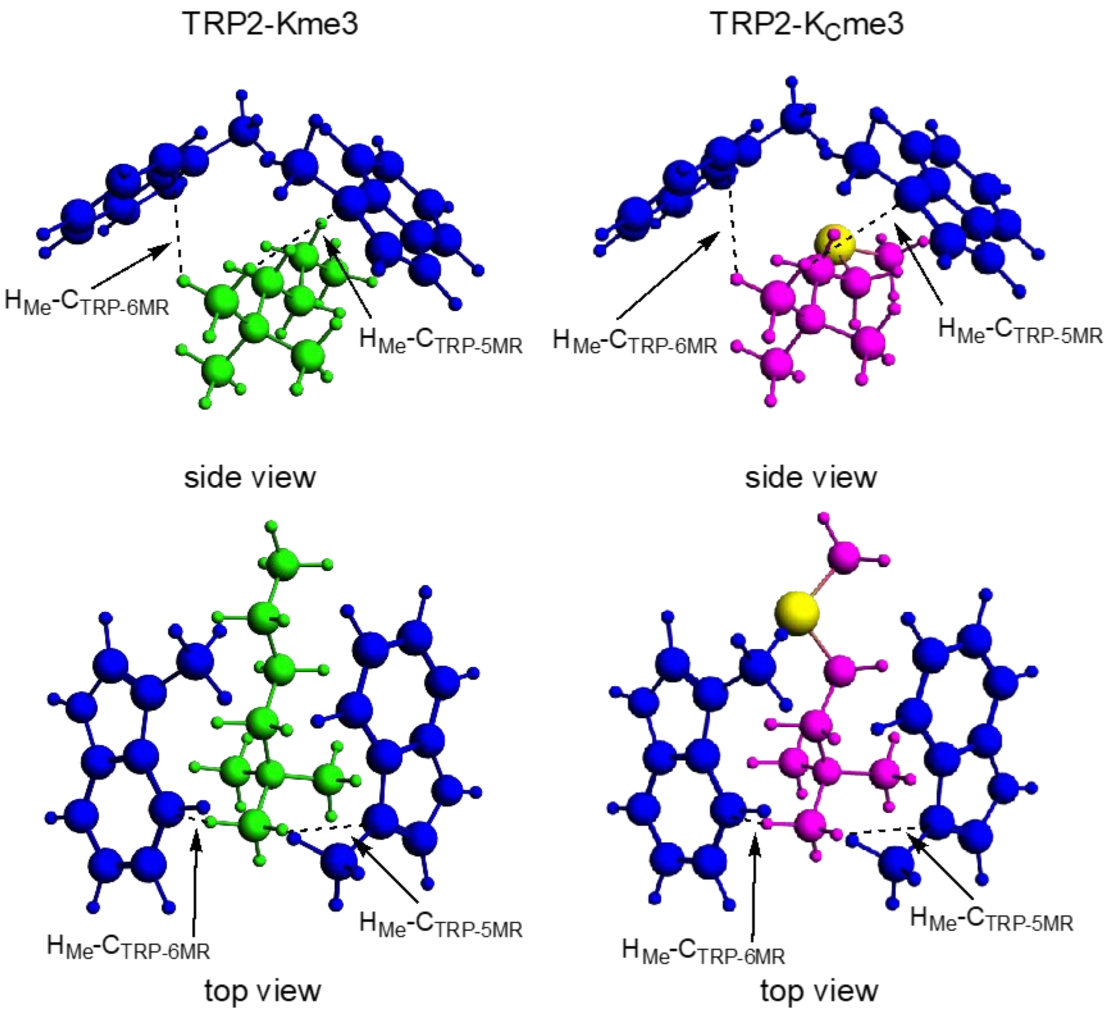

Figure S7 Top view of structure of TRP2-Kme3 and TRP-Kcme3 model complexes. TRP2 in blue, Kme3 in green and Kcme3 in pink (except S atom in yellow).

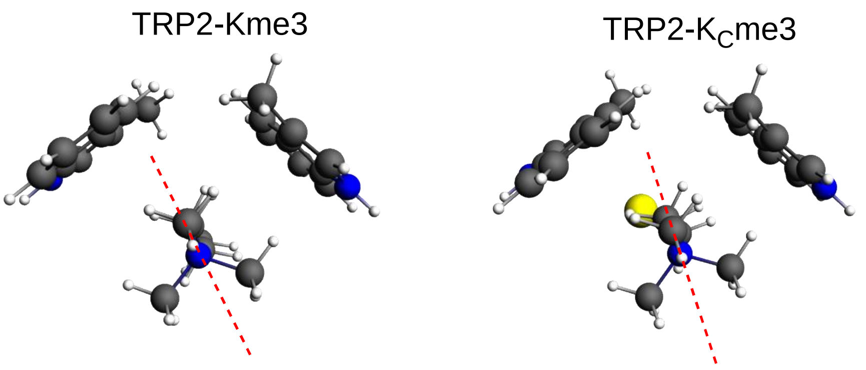

Figure S8 Front view of the structure of TRP-Kme3 and TRP2-Kcme3 model complexes. The plane till C^{β} is indicated by a red dotted line.

Table S5. Overlaps between the MOs of TRP and Kme3 or Kcme3.[a].

TRP2 MOs	Kme3/ Kcme3 MOs	TRP2-Kme3	TRP2-Kcme3
HOMO	LUMO	0.012	0.019
HOMO	LUMO+1	0.006	0.012
**HOMO-1**	**LUMO**	0.028	0.024
HOMO-1	LUMO+1	0.006	0.011

[a] Computed at BLYP-D3BJ/TZ2P.

Table S6. Cartesian coordinates (in Å) of TRP2-Kme3 and TRP2-Kcme3complexes, computed at BLYP-D3BJ/TZ2P using COSMO to simulate aqueous solvation and a constrained optimization to simulate the effect of the protein backbone.

**TRP2-Kme3:**			
C	-14.114000000	-20.049000000	-0.875000000
C	-14.962000000	-19.738000000	0.323000000
C	-15.235000000	-20.561000000	1.377000000
C	-15.571000000	-18.476000000	0.628000000
C	-16.191000000	-18.610000000	1.893000000
C	-15.649000000	-17.250000000	-0.044000000
N	-15.971000000	-19.886000000	2.326000000
C	-16.882000000	-17.550000000	2.500000000
C	-16.335000000	-16.198000000	0.561000000
C	-16.943000000	-16.358000000	1.823000000
H	-17.473000000	-15.517000000	2.270000000
H	-14.000000000	-19.128000000	-1.447000000
H	-14.917000000	-21.601000000	1.456000000
H	-15.183000000	-17.121000000	-1.021000000
H	-16.295000000	-20.273000000	3.201000000
H	-17.354000000	-17.669000000	3.475000000
H	-16.402000000	-15.237000000	0.051000000
H	-13.186000000	-20.452000000	-0.470000000
C	-13.008000000	-14.944000000	-1.752000000
C	-11.604000000	-15.279000000	-1.421000000
C	-10.629000000	-14.423000000	-0.994000000
C	-10.999000000	-16.571000000	-1.507000000
C	-9.651000000	-16.428000000	-1.114000000
C	-11.469000000	-17.840000000	-1.880000000
N	-9.451000000	-15.109000000	-0.805000000
C	-8.764000000	-17.507000000	-1.084000000
C	-10.588000000	-18.912000000	-1.851000000
C	-9.247000000	-18.738000000	-1.453000000
H	-8.579000000	-19.599000000	-1.438000000
H	-13.651000000	-15.747000000	-1.391000000
H	-10.764000000	-13.354000000	-0.828000000
H	-12.506000000	-17.981000000	-2.186000000
H	-8.581000000	-14.705000000	-0.490000000
H	-7.726000000	-17.376000000	-0.779000000
H	-10.938000000	-19.903000000	-2.140000000
H	-13.236000000	-13.992000000	-1.272000000
H	-14.522374582	-20.815706247	-1.547885097
H	-13.158423449	-14.818069475	-2.834003080
C	-10.114752602	-21.305220763	1.892377384
C	-11.216790553	-20.285805453	1.565697139
C	-11.002507694	-18.940388922	2.287874794
C	-12.090102444	-17.946438531	1.883398917
N	-12.070579136	-16.609009105	2.645799587
C	-13.150209827	-15.721875154	2.061139245

C	-10.731973677	-15.916856909	2.492497579
C	-12.365551231	-16.823749860	4.115733524
H	-12.408951734	-15.846708144	4.598931529
H	-9.130774938	-20.927740553	1.584711029
H	-12.198761989	-20.691389652	1.844793551
H	-10.013332154	-18.548608026	2.021388165
H	-13.089556823	-18.360771942	2.048366284
H	-12.918787327	-15.550085307	1.009793743
H	-10.798479096	-14.941374934	2.976931329
H	-11.570405162	-17.423783848	4.555007649
H	-10.074709060	-21.512398227	2.969877086
H	-11.238229637	-20.105747697	0.484787617
H	-11.011890000	-19.111207919	3.371339633
H	-11.995495315	-17.683123359	0.825941434
H	-13.153012338	-14.778635478	2.609325685
H	-10.522561113	-15.800602201	1.428638506
H	-13.326128627	-17.334212341	4.203429545
H	-14.111323996	-16.226695834	2.162298263
H	-9.963428736	-16.520728269	2.972373589
H	-10.290718944	-22.253949886	1.371226478

**TRP2-Kcme3**			
C	-14.114000	-20.049000	-0.875000
C	-14.962000	-19.738000	0.323000
C	-15.235000	-20.561000	1.377000
C	-15.571000	-18.476000	0.628000
C	-16.191000	-18.610000	1.893000
C	-15.649000	-17.250000	-0.044000
N	-15.971000	-19.886000	2.326000
C	-16.882000	-17.550000	2.500000
C	-16.335000	-16.198000	0.561000
C	-16.943000	-16.358000	1.823000
H	-17.473000	-15.517000	2.270000
H	-14.000000	-19.128000	-1.447000
H	-14.917000	-21.601000	1.456000
H	-15.183000	-17.121000	-1.021000
H	-16.295000	-20.273000	3.201000
H	-17.354000	-17.669000	3.475000
H	-16.402000	-15.237000	0.051000
H	-13.186000	-20.452000	-0.470000
C	-13.008000	-14.944000	-1.752000
C	-11.604000	-15.279000	-1.421000
C	-10.629000	-14.423000	-0.994000
C	-10.999000	-16.571000	-1.507000
C	-9.651000	-16.428000	-1.114000
C	-11.469000	-17.840000	-1.880000
N	-9.451000	-15.109000	-0.805000
C	-8.764000	-17.507000	-1.084000

C	-10.588000	-18.912000	-1.851000
C	-9.247000	-18.738000	-1.453000
H	-8.579000	-19.599000	-1.438000
H	-13.651000	-15.747000	-1.391000
H	-10.764000	-13.354000	-0.828000
H	-12.506000	-17.981000	-2.186000
H	-8.581000	-14.705000	-0.490000
H	-7.726000	-17.376000	-0.779000
H	-10.938000	-19.903000	-2.140000
H	-13.236000	-13.992000	-1.272000
H	-14.522375	-20.815706	-1.547885
H	-13.158423	-14.818069	-2.834003
C	-10.114753	-21.305221	1.892377
S	-11.751641	-20.485915	1.925294
C	-11.154204	-18.746106	2.097325
C	-12.327957	-17.795118	1.855623
N	-12.210288	-16.468116	2.621354
C	-13.282495	-15.531344	2.104758
C	-10.853986	-15.829722	2.402710
C	-12.440562	-16.698791	4.101531
H	-12.360513	-15.738277	4.611913
H	-9.539417	-20.959707	1.027526
H	-10.084953	-16.473255	2.827641
H	-10.363883	-18.579007	1.359042
H	-13.280929	-18.226877	2.172272
H	-13.086364	-15.335803	1.050540
H	-10.852574	-14.862788	2.907568
H	-11.685275	-17.387599	4.477981
H	-9.561888	-21.107338	2.815931
H	-10.299346	-22.379217	1.802303
H	-10.732360	-18.635435	3.099951
H	-12.397931	-17.537395	0.797294
H	-13.232898	-14.606600	2.680555
H	-10.704050	-15.701361	1.330842
H	-13.439966	-17.117410	4.229877
H	-14.254367	-16.009384	2.228445

6. LC-MS of purified histone peptides

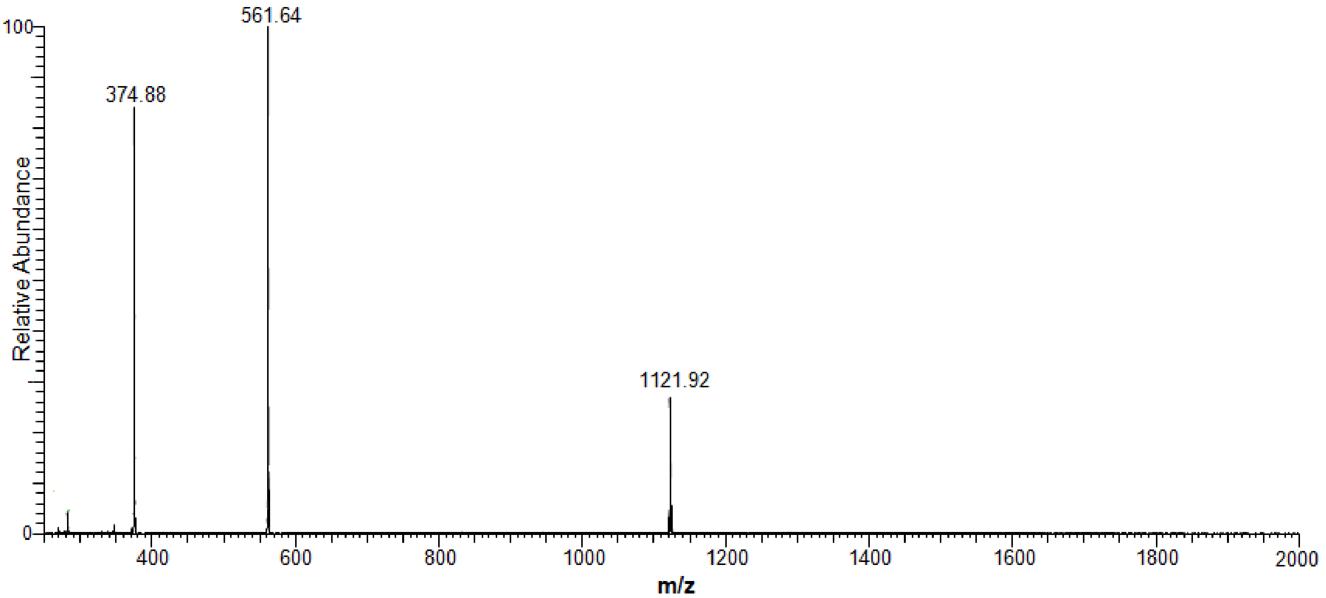

Figure S9. LC-MS analysis of 1-10 H3C4 after RP-HPLC purification.

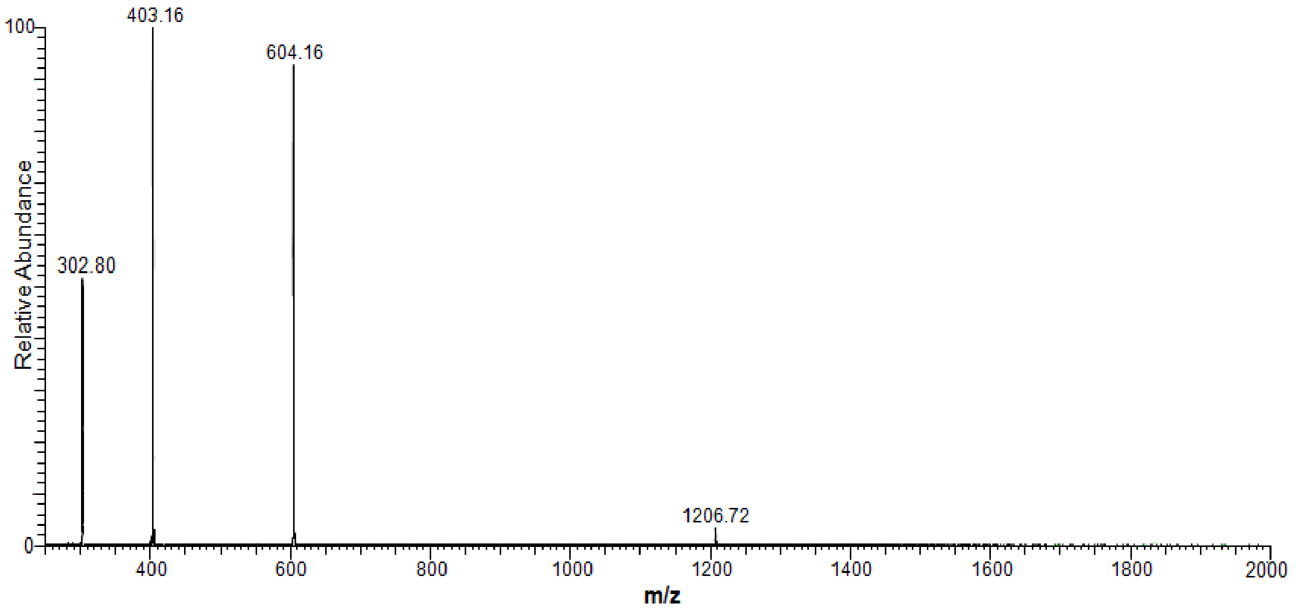

Figure S10. LC-MS analysis of 1-10 H3Kc4me3 after RP-HPLC purification.

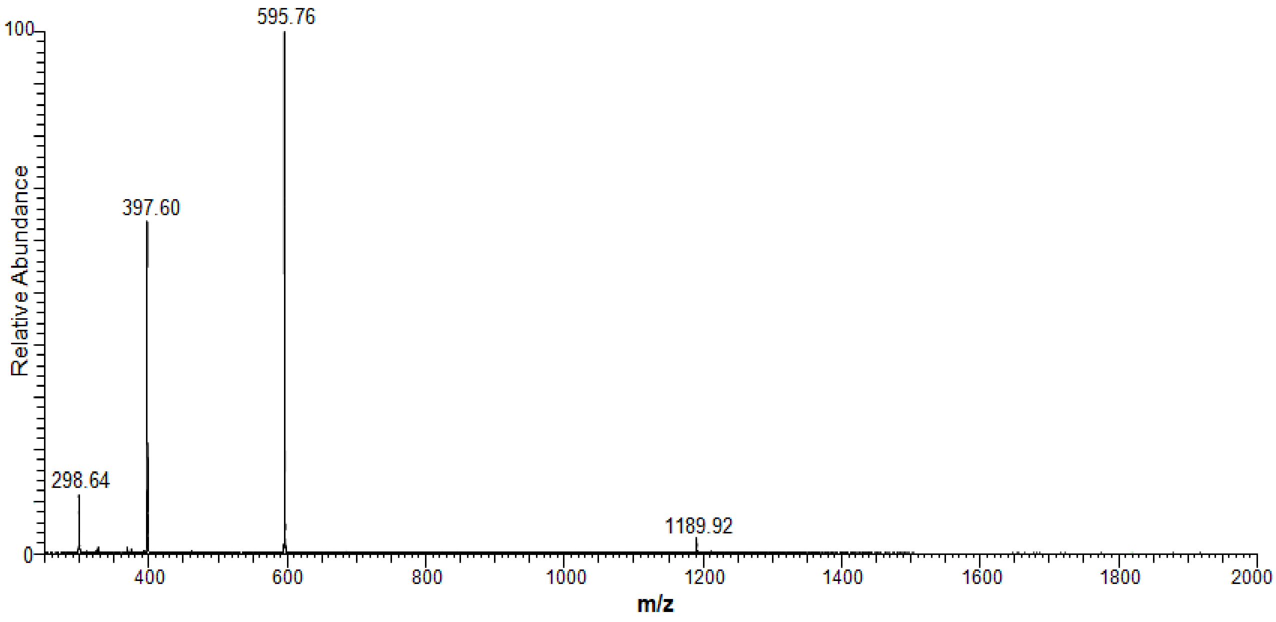

Figure S11. LC-MS analysis of 1-10 H3K4me3 after RP-HPLC purification.
